# A prediction model for predicting relapsed-free survival of early-stage invasive breast cancer patients with hormone receptor positive based on Ki67, HER2 and TOP2A

**DOI:** 10.3389/fonc.2025.1552937

**Published:** 2025-04-09

**Authors:** Dawei Yuan, Rulan Ma, Haixia Ye, Wenbo Liu

**Affiliations:** ^1^ Department of Surgical Oncology, The First Affiliated Hospital of Xi’an Jiaotong University, Xi’an, China; ^2^ The Second Clinical College, Department of Medicine, Wuhan University, Wuhan, China; ^3^ Department of Plastic and Cosmetic Maxillofacial Surgery, The First Affiliated Hospital of Xi’an Jiaotong University, Xi’an, Shannxi, China

**Keywords:** invasive breast cancer, hormone receptor positive, relapse-free survival, TOP2A, nomogram

## Abstract

**Objective:**

The purpose of the current study was to determine the relationship between ribonucleotide reductase M1 (RRM1), topoisomerase II alpha (TOP2A), Thymidylate synthase (TYMS), class III beta-tubulin (TUBB3) and phosphatase and tensin homolog (PTEN) expressions and relapse-free survival (RFS) in early-stage invasive breast cancer (IBC) patients with hormone receptor positive (HR^+^), as well as to develop a nomogram model for forecasting RFS.

**Methods:**

Early-stage IBC patients with HR^+^ who were diagnosed and treated at the First Affiliated Hospital of Xi’an Jiaotong University from June 2017 to December 2020 were enrolled in this study. The survival analysis was performed by utilizing the Kaplan-Meier method, and the risk factors linked to patient RFS were determined by performing Cox regression analysis. The nomogram for predicting RFS in early-stage IBC patients with HR^+^ was stablished and validated based on the results of the Cox regression analysis.

**Results:**

In total, 126 early-stage IBC patients with HR^+^ were included in the current study. Among these patients, 23 cases experienced relapse after surgery, with a median RFS of 29 months. Significant relationships were observed between TYMS, RRM1, TUBB3, TOP2 and PTEN, Ki67 and human epidermal growth factor receptor 2 (HER2) and patient RFS. Cox regression analysis revealed that chemotherapy and higher expression levels of TOP2A, HER2 and Ki67 were independent predictors of RFS in early-stage IBC patients with HR^+^. The nomogram we constructed using the above independent risk factors exhibited good ability for predicting RFS in early-stage IBC patients with HR^+^.

**Conclusion:**

Chemotherapy, TOP2A, HER2, and Ki67 expression were independent predictors of RFS in early-stage IBC patients with HR^+^. The nomogram we developed using these predictors is a reliable tool for predicting RFS in this patient population.

## 1 Introduction

Globally, breast cancer (BC) has become the most common malignancy and a significant public health burden (1). The incidence of BC accounts for approximately 12.5% of new cancer cases, and more than 6 million women worldwide die from BC each year (https://www.wcrf.org/cancer-trends/worldwide-cancer-data/). The incidence of BC has gradually increased due to an aging population and some screening methods, but the survival rate of BC has also improved significantly in the past few decades. A recent study has shown that patients with early BC recurrence have a worse prognosis than those with late recurrence (2). Hormone receptor (HR)-negative (HR^-^) BC patients have a higher risk of recurrence in the first two years after initial diagnosis, but their risk of recurrence has declined rapidly thereafter, lower than HR-positive (HR^+^) patients (3, 4). Patients with HR^+^ BC have an annual risk of recurrence that stays unchanged for a long time, and recurrence may occur within ten years after the initial diagnosis (3). The prognosis of early recurrence is worse than that of late recurrence (5).

It has been reported that the detection of multiple gene expressions, including ribonucleotide reductase M1 (RRM1), topoisomerase II alpha (TOP2A), class III beta-tubulin (TUBB3), Thymidylate synthase (TYMS), can guide individualized chemotherapy to improve therapeutic efficacy and reduce side effects in BC patients (6).Additionally, gene expressions of RRM1, TOP2A, TYMS and TUBB3 have been linked to objective treatment response in BC patients (7). TYMS is a key target gene of 5-fluorouracil in BC patients (8), and increased TYMS expression is associated with a poor prognosis (9). RRM1 expression significantly correlates with sensitivity to gemcitabine and is related to estrogen receptor (ER) status, lymph node metastasis and pathological classification (10). TUBB3 serves as a biomarker for resistance of docetaxel and paclitaxel, while TOP2A expression level obviously is related to anthracyclines efficacy (11). TUBB3 and TOP2A also serve as critical prognostic factors for predicting overall survival (12). It is reported that high TUBB3 expression is associated with low response for taxanes chemotherapy and tumor grade, whereas low TUBB3 and TOP2A expression is associated with improved clinical outcomes (12–14). Individuals with both high human epidermal growth factor receptor 2 (HER2) and TOP2A protein expression present a poor prognosis in T1N0 BC patients and can benefit from anthracycline-based regimens (14). Loss of phosphatase and tensin homolog (PTEN) activity is associated with multiple primary and metastatic malignancies, including BC (15). PTEN serves as a crucial indicator for detecting the occurrence, development, invasion and metastasis of BC, and the loss of PTEN expression may result in lymph node metastasis of BC (16). Therefore, it can serve as an objective indicator toassess the biological behavior of BC.

However, it is still unclear whether RRM1, TOP2A, TYMS, TUBB3 and PTEN can be utilized to prognosticate relapse-free survival (RFS) in early-stage invasive BC (IBC) patients with HR^+^. Therefore, the aim of this research was to examine the relationship between the aforementioned genes and RFS in IBC patients. Ultimately, a nomogram model was created to predict RFS in early-stage IBC patients with HR^+^.

## 2 Methods

### 2.1 Study population

In total, 126 IBC patients with HR^+^ who were diagnosed and treated in the First Affiliated Hospital of Xi’an Jiaotong University between June 2017 and December 2020 were enrolled in this research. The inclusion criteria are as follows: 1) received surgery; 2) operative pathological diagnosis indicated early-stage IBC with HR^+^ (stage I/II); 3) the availability of gene expression data for Ki67, TYMS, RRM1, TUBB3, TOP2A, HER2 and PTEN; 4) complete clinicopathological data. The exclusion criteria are as follows: 1) surgery was not performed; 2) inability to obtain gene expression data; 3) incomplete clinicopathological data. This study adhered to the Declaration of Helsinki, and was approved and supervised by the Ethics Committee of the First Affiliated Hospital of Xi’an Jiaotong University (No. XJTU1AF2022LSK-335).

### 2.2 Data collection and processing

The expression levels of the genes (Ki67, TYMS, RRM1, TUBB3, TOP2A, HER2 and PTEN) in IBC patients were tested by Surexam Co., Ltd (Guangzhou, China). The clinicopathological data, treatment-related data, along with the gene expression data of the patients, were collected for further analysis. Data processing was carried out undoing Microsoft Excel and SPSS26.0 software. The receiver operating characteristic (ROC) curve was used to determine the optimal cut-off value of the continuous data. Following this, the continuous variables were transformed into binary variables based on the cut-off values.

### 2.3 Construction and validation of the nomogram prediction model

Cox regression analysis was utilized to calculate Hazard ratios (HRs) and 95% confident intervals (CIs) for the variables. According to the results of univariate Cox regression analysis, factors with P<0.05 in the were selected for further multivariate Cox regression analysis to identify independent factors related to the RFS of IBC patients with HR^+^. Concordance-index (C-index), area under the curve (AUC) of the ROC and Bootstrap calibration curve were conducted to assess the discrimination and calibration of the nomogram.

### 2.4 Statistical analysis

SPSS 26.0 software was used for data analysis. The difference between the two groups was analyzed by Chi-square test. Survival analysis was conducted by using Kaplan-Meier method, and the Log-rank test was used to analyze the survival difference between two groups. The factors related to the RFS of the patients were determined by Cox regression analysis. Development and validation of the nomogram for forecasting RFS were conducted by RStudio software. P<0.05 was considered to be statistically significant.

## 3 Results

### 3.1 Characteristics of early-stage IBC patients with HR^+^

126 patients were enrolled in this research according to the inclusion and exclusion criteria. The medium follow-up time was 27 months. Among these patients, 23 cases relapsed after surgery, with a median RFS of 29 months. From the **Table 1**, we observed that patient age (P=0.009), tumor grade (P=0.032), tumor diameter (P=0.020), sentinel lymph node (P=0.014), Ki67 expression (P<0.001), chemotherapy (P<0.001), and the expression levels of TYMS (P=0.020), RRM1 (P=0.010), TOP2A (P<0.001), HER2 (P=0.004) and PTEN (P<0.001) were associated with the relapse of early-stage IBC patients with HR^+^.

**Table 1:** Baseline data of early-stage IBC patients with HR^+^.

Factors		Non-relapse (N=103)	Relapse (N=23)	Total	X^2^	P
**Age (Years)**	<37.5	6	6	12		**0.009** ^a^
	≥37.5	97	17	114		
**Pathological type**	NSIDC	74	20	94		0.154^a^
	ILC	12	0	12		
	NSIDC-CIS	11	1	12		
	CIS-MI	2	1	3		
	SGDC	0	1	1		
	CC	2	0	2		
	MC	2	0	2		
**Grade**	1	2	1	3		**0.032** ^a^
	2	54	8	62		
	3	20	10	30		
	NA	31				
**Tumor diameter (cm)**	<16	41	2	43	7.354	**0.010**
	≥16	60	19	79		
	NA	4				
**Menopausal status**	No	44	10	54	0.038	1.000
	Yes	39	8	47		
	NA	25				
**AJCC stage**	I	52	8	60	0.173	0.248
	II	51	15	66		
**Sentinel lymph node**	<7	75	9	84	1.859	**0.014**
	≥7	25	11	36		
	NA	6				
**Ki67**	<22.50%	89	9	98	24.314	**0.000**
	≥22.50%	14	14	28		
**Chemotherapy**	No	81	9	90	14.382	**0.000**
	Yes	22	14	36		
**Endocrine therapy**	Anastrozole	2	1	3		0.209^a^
	Aletrozole	52	9	61		
	Tamoxifen	47	12	59		
	Toremifen	1	0	1		
	Exemestane	0	1	1		
	NA	1				
**TYMS**	<56.75%	64	8	72	5.744	**0.020**
	≥56.75%	39	15	54		
**RRM1**	<68.80%	79	11	90	7.680	**0.010**
	≥68.80%	24	12	36		
**TUBB3**	<39.60%	46	5	51	4.100	0.059
	≥39.60%	57	18	75		
**TOP2A**	<63.35%	82	6	88	25.573	**0.000**
	≥63.35%	21	17	38		
**HER2**	<65.25%	74	14	88	8.951	**0.004**
	≥65.25%	29		29		
**PTEN**	<46.75%	25	16	41	17.571	**0.000**
	≥46.75%	78	7	85		

a Fisher exact probability method. IBC, invasive breast cancer; HR, hormonal receptor; HER2, human epidermal growth factor receptor 2; TYMS, thymidylate synthase; RRM1, ribonucleotide reductase M1; TUBB3, class III beta-tubulin; TOP2A, topoisomerase II alpha; PTEN, phosphatase and tensin homolog; NSIDC, non-specific invasive ductal carcinoma; ILC, invasive lobular carcinoma; NSIDC-CIS, non-specific invasive ductal carcinoma with carcinoma *in situ*; CIS-MI, carcinoma *in situ* with microinvasion; SGDC, sweat gland differentiated carcinoma; CC, cribriform carcinoma; MC, mucoid carcinoma; AJCC, American Joint Committee on Cancer; NA, not applicable. The bolded values present P<0.05.

### 3.2 Cox regression analysis for identifying the factors related to the RFS of early-stage IBC patients with HR^+^

**Figure 1:**
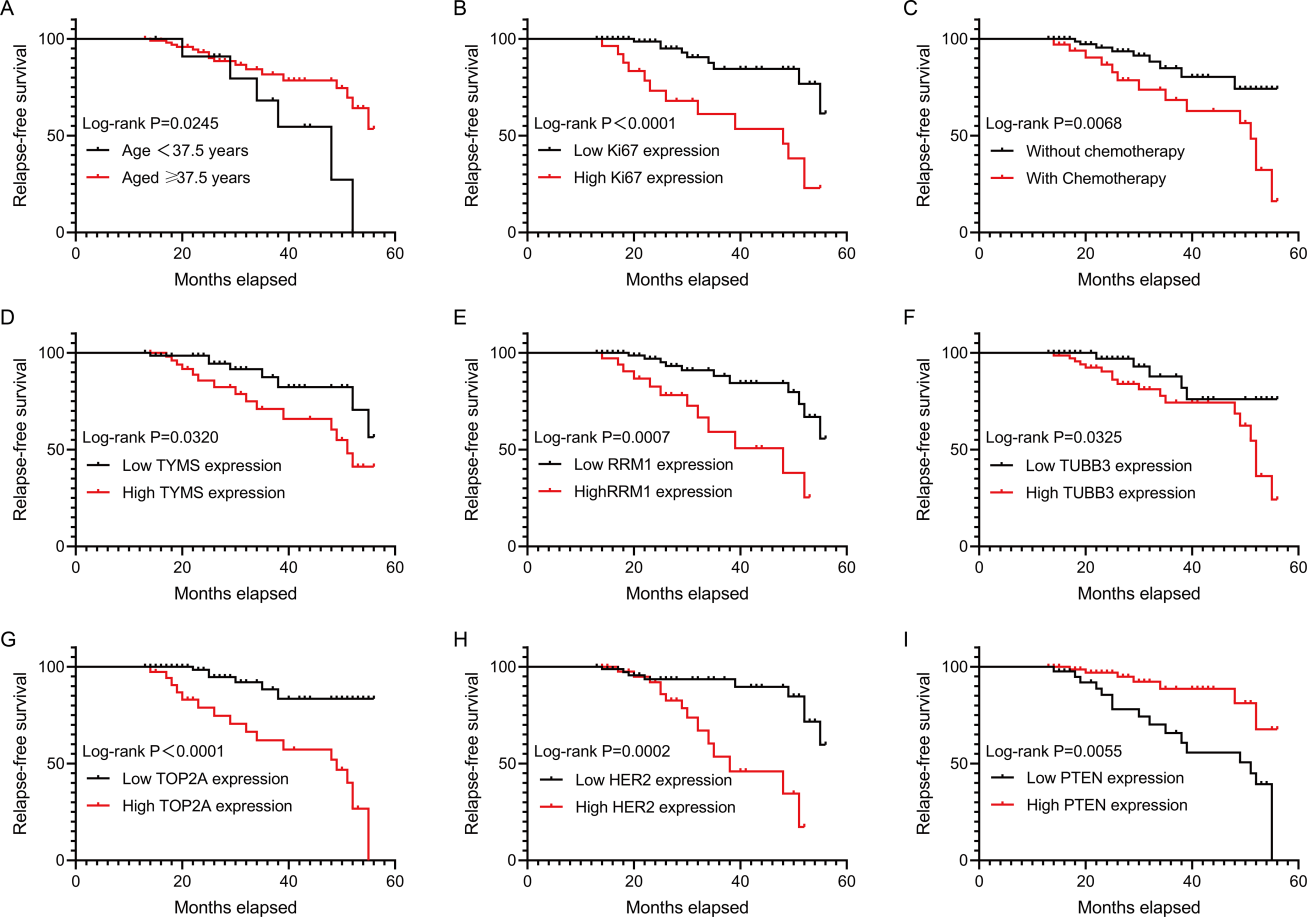
RFS analysis of early-stage IBC patients with HR^+^. The correlations of RFS of HR^+^ early-stage IBC patients with age, Ki67 expression, chemotherapy, TYMS expression, RRM1 expression, TUBB3 expression, TOP2A expression, HER2 expression and PTEN expression were analyzed by using Kaplan-Meier method. RFS, relapse-free survival; IBC, invasive breast cancer; HR, hormonal receptor; TYMS, thymidylate synthase; RRM1, ribonucleotide reductase M1; TUBB3, class III beta-tubulin; TOP2A, topoisomerase II alpha; PTEN, phosphatase and tensin homolog; HER2, human epidermal growth factor receptor 2.

To determine the factor that might affect patient relapse after surgery, we conducted univariate Cox regression analysis. The results showed that patient age (HR: 0.354, 95%CI: 0.137-0.914, P=0.032), Ki67 expression (HR: 4.684, 95%CI: 1.989-11.033, P<0.001), chemotherapy (HR:3.023, 95%CI: 1.299-7.033, P=0.010), the expressions of TYMS (HR: 2.507, 95%CI: 1.049-5.991, P=0.039), RRM1 (HR: 3.858, 95%CI: 1.658-8.979, P=0.002), TUBB3 (HR: 2.819, 95%CI: 1.041-7.629, P=0.041), TOP2A (HR:6.021, 95%CI: 2.342-15.480, P<0.001), HER2 (HR: 0.200, 95%CI: 0.080-0.501, P=0.001) and PTEN (HR: 0.270, 95%CI: 0.109-0.666, P=0.004) were significantly related to the RFS of the patients (**Table 2**). Moreover, survival analyses used by Kaplan-Meier method also showed that the above factors were correlated with the RFS of early-stage IBC patients with HR^+^ (**Figure 1**). Thus, these factors were enrolled in further multivariate Cox regression analysis. We observed that chemotherapy (HR: 3.601, 95%CI: 1.395-9.298, P=0.008) and the expression levels of Ki67 (HR: 3.143, 95%CI: 1.143-8.647, P=0.027), TOP2A (HR: 3.331, 95%CI: 1.053-10.531, P=0.041) and HER2 (HR: 7.501, 95%CI: 2.674-21.036, P<0.001) were the independent factors affecting the RFS of the patients (**Table 3**).

**Table 2:** Univariate Cox regression analysis for relapse of early-stage IBC patients with HR^+^.

Factors		Univariate analysis			
		HR	95%CI		P
			Lower	Upper	
**Age (Years)**	<37.5 vs ≥37.5	0.354	0.137	0.914	**0.032**
**Pathological type**	NSIDC vs ILC vs NSIDC-CIS vs CIS-MI vs SGDC vs CC vs MC	0.849	0.553	1.305	0.456
**Grade**	1 vs 2 vs 3	2.375	1.001	5.632	0.050
**Tumor diameter (cm)**	<16 vs ≥16	4.216	0.978	18.177	0.054
**Menopausal status**	No vs Yes	1.022	0.401	2.601	0.964
**AJCC stage**	I vs II	2.092	0.881	4.964	0.094
**Sentinel lymph node**	<7 vs ≥7	2.362	0.975	5.725	0.057
**Ki67**	<22.50% vs ≥22.50%	4.684	1.989	11.033	**0.000**
**Chemotherapy**	No vs Yes	3.023	1.299	7.033	**0.010**
**Endocrine therapy**	Anastrozole vs Aletrozole vs Tamoxifen vs Toremifen vs Exemestane	1.010	0.487	2.096	0.979
**TYMS**	<56.75% vs ≥56.75%	2.507	1.049	5.991	**0.039**
**RRM1**	<68.80% vs ≥68.80%	3.858	1.658	8.979	**0.002**
**TUBB3**	<39.60% vs ≥39.60%	2.819	1.041	7.629	**0.041**
**TOP2A**	<63.35% vs ≥63.35%	6.021	2.342	15.480	**0.000**
**HER2**	<65.25% vs ≥65.25%	0.200	0.080	0.501	**0.001**
**PTEN**	<46.75% vs ≥46.75%	0.270	0.109	0.666	**0.004**

IBC, invasive breast cancer; HR, hormonal receptor; HER2, human epidermal growth factor receptor 2; TYMS, thymidylate synthase; RRM1, ribonucleotide reductase M1; TUBB3, class III beta-tubulin; TOP2A, topoisomerase II alpha; PTEN, phosphatase and tensin homolog; NSIDC, non-specific invasive ductal carcinoma; ILC, invasive lobular carcinoma; NSIDC-CIS, non-specific invasive ductal carcinoma with carcinoma *in situ*; CIS-MI, carcinoma *in situ* with microinvasion; SGDC, sweat gland differentiated carcinoma; CC, cribriform carcinoma; MC, mucoid carcinoma; AJCC, American Joint Committee on Cancer; HR, hazard ratio; CI, confidence interval. The bolded values present P<0.05.

**Table 3:** Multivariate Cox regression analysis for early-stage IBC patients with HR^+^.

Factors		Multivariate analysis			
		HR	95%CI		P
			Lower	Upper	
**Ki67**	<22.50% vs ≥22.50%	3.601	1.395	9.298	**0.008**
**Chemotherapy**	No vs Yes	3.143	1.143	8.647	**0.027**
**TOP2A**	<63.35% vs ≥63.35%	3.331	1.053	10.531	**0.041**
**HER2**	<65.25% vs ≥65.25%	7.501	2.674	21.036	**0.000**

IBC, invasive breast cancer; HR, hormonal receptor; HER2, human epidermal growth factor receptor 2; TOP2A, topoisomerase II alpha; HR, hazard ratio; CI, confidence interval. The bolded values present P<0.05.

### 3.3 Construction and validation of the nomogram for predicting the RFS of early-stage IBC patients with HR^+^

**Figure 2:**
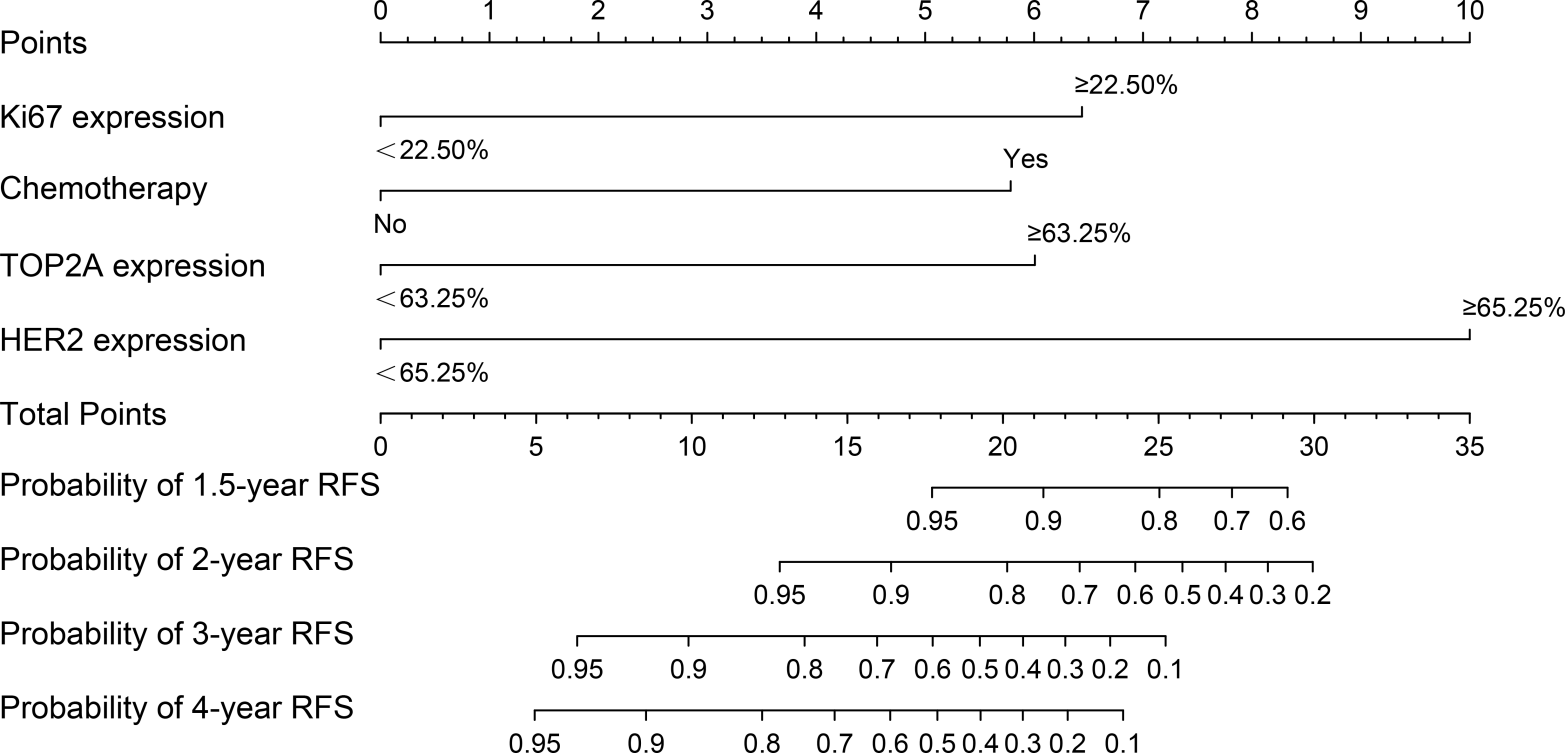
The nomogram for predicting RFS of early-stage IBC patients with HR^+^. RFS, relapse-free survival; IBC, invasive breast cancer; HR, hormonal receptor; TOP2A, topoisomerase II alpha; HER2, human epidermal growth factor receptor 2.

**Figure 3:**
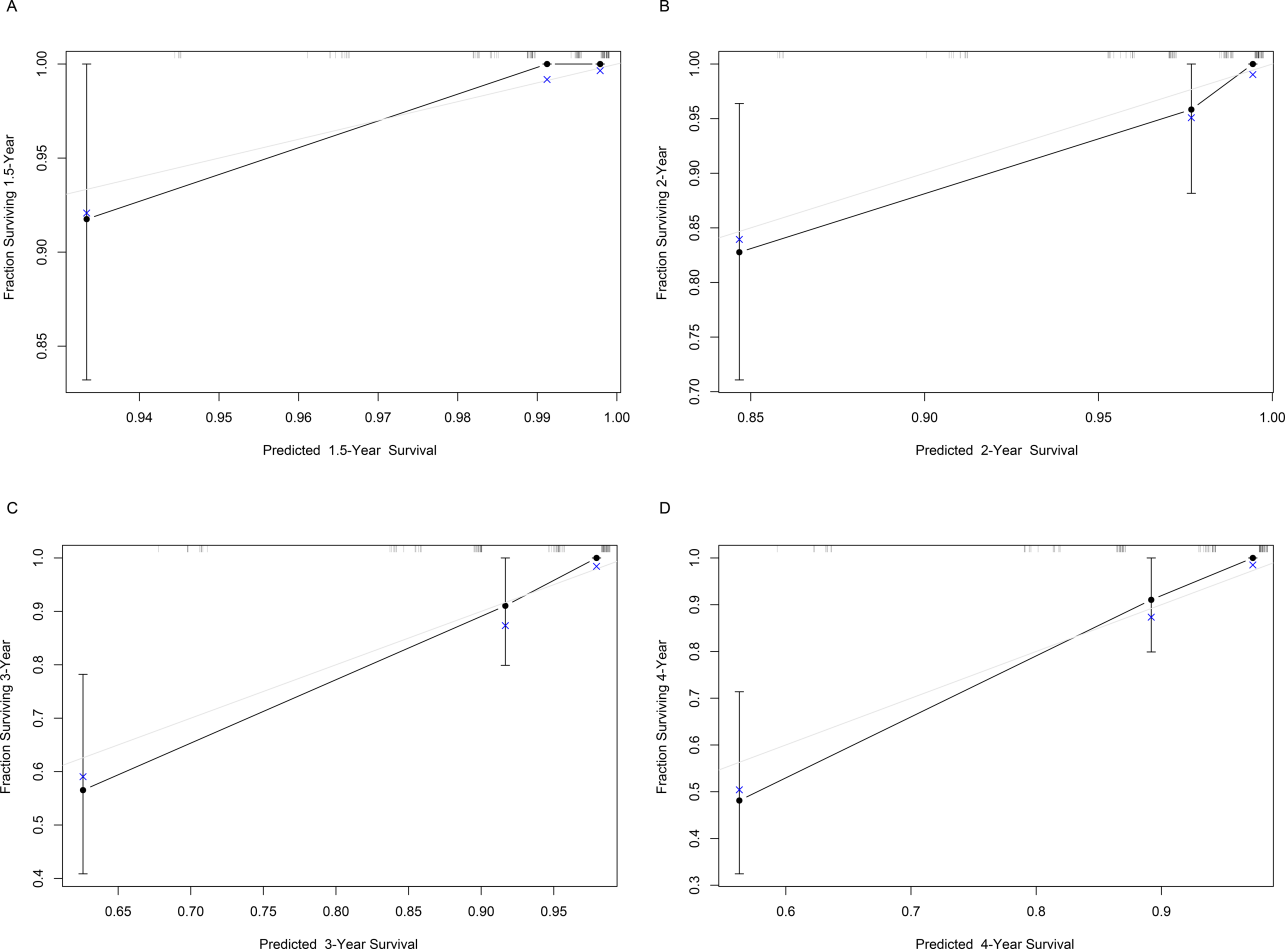
Bootstrap calibration curve of the nomogram for predicting RFS of early-stage IBC patients with HR^+^. The X-axis represents the predicted probability, and the Y-axis represents the actual probability. The gray line represents the ideal value, and the black line represents the predicted value. It is showed that the trend of the predicted value is consistent with the true value, indicating the good calibration effect of the nomogram. RFS, relapse-free survival; IBC, invasive breast cancer; HR, hormonal receptor.

Based on Ki67 expression, chemotherapy, TOP2A expression and HER2 expression, we developed a nomogram for predicting RFS (**Figure 2**). The c-index of the nomogram was 0.833, indicating the nomogram had good discrimination. Besides, the Bootstrap calibration curve also indicated that the trend between true values and predicted values was consistent, suggesting that the nomogram had the accuracy for forecasting RFS of early-stage IBC patients with HR^+^ (**Figure 3**).

## 4 Discussion

Although the risk of recurrence and death was decreased after adjuvant endocrine therapy, up to 20% of early BC patients will experience relapse with 10 years after surgery, including local and distant metastasis. Hence, it is imperative to investigate the risk factor for early-stage IBC patients and establish a relevant prediction model.

Previous studies have showed that RRM1, TOP2A, TYMS, TUBBS, and PTEN are prognostic markers and can reflect patients’ response to treatment in BC (6, 7, 16). In the current study, the predictive role of RRM1, TOP2A, TYMS, TUBB3 and PTEN in RFS of early-stage IBC with HR^+^, and other possible factors associated with RFS were investigated. Our findings indicated that chemotherapy, TOP2A, HER2 and Ki67 were independent predictors of RFS. The nomogram is a convenient tool for forecasting the outcome, which is widely used to quantify risk in various disease (17). Thus, based on the above independent risk factors (chemotherapy, TOP2A, HER2 and Ki67), we established a nomogram for predicting RFS. The c-index of the nomogram indicated a good discrimination, while the Bootstrap calibration curve indicated that the nomogram had accuracy for predicting RFS.

TOP2A is a gene that encodes enzyme topoisomerase IIα, which can break down DNA supercoils through reversible double-stranded DNA breaks during DNA replication and transcription. The enzyme topoisomerase IIα is considered to be the target of anthracyclines. Anthracycline drugs stabilize double-stranded DNA breakage by binding to enzyme topoisomerase IIα, ultimately leading to apoptosis. It has been reported that TOP2A amplification occurs in 2-9% of BC cases (18, 19). Moreover, in patients receiving anthracycline-based adjuvant therapy, TOP2A amplification has a significant impact on RFS and overall survival (18, 20). BC patients with TOP2A amplification showed a better response to adjuvant chemotherapy and a better prognosis (21), while patients with TOP2A deletion showed resistance to anthracyclines (19). There is also a significant association between TOP2A amplification and recurrence in BC patients, including ER^+^ BC (22, 23). In this research, we observed that TOP2A was an independent predictor for RFS, and the increased expression of TOP2A was related to the increased risk of recurrence in early-stage IBC patients with HR^+^, which was consistent with the findings of previous studies (12, 14, 22–24). Thus, it is indicated that TOP2A expression can provide prognostic information in HR^+^ early-stage IBC and may be used to identify patients with high risk of recurrence.

One of the characteristics of malignant tumors is a high proliferation rate (25). Therefore, a high proliferation rate is also an critical variable of tumor prognosis. As a classic proliferation marker, Ki67 has been found to be significantly related to the prognosis, including RFS, of BC patients (24, 26). High Ki67 level is associated with poor prognosis and chemotherapy response (24, 27–29). Similarly, our results showed that higher Ki67 expression was related to worse RFS and was an independent predictor for relapse of HR^+^ early-stage IBC patients. Besides, we found that patients with higher HER2 level had significantly improved RFS, and HER2 expression was independently relevant to the recurrence of the patients, which is consistent with the previous findings (30). These findings indicated that combined detection of these independent risk factors might provide the clinicians with a simple and convenient tool for predicting the probability of recurrence in early-stage IBC patients with HR^+^.

However, the limitations in this study cannot be ignored. First, as a single-center and retrospective research, the sample size was relatively small, and only internal validation of the nomogram was performed. Second, only the expression levels of the target genes and some common clinicopathological indexes were analyzed in this study, the laboratory indicators and other potential predictors of the patients were not included for analyzing. Third, although the population of this study was included by inclusion and exclusion criteria, the impact of individual factors on RFS cannot be avoided. Therefore, further studies should be conducted using a prospective, multi-center approach with a larger sample size to verify the findings of the current study. Additionally, external validation of the nomogram should be performed based on a multi-center study.

## 5 Conclusion

Our study revealed that chemotherapy, TOP2A, HER2 and Ki67 were independent predictors for RFS in HR^+^ early-stage IBC patients. The nomogram constructed in this research showed good discrimination and accuracy in predicting RFS of the patients. This provides a convenient tool to predict the recurrence probability in early-stage IBC patients with HR^+^.

## Data Availability

The original contributions presented in the study are included in the article/supplementary material. Further inquiries can be directed to the corresponding author.
